# Supplementary material for: Factors and impact of physicians’ diagnostic errors in malpractice claims in Japan
Source: PLoS One. 2020 Aug 3;15(8):e0237145. doi: 10.1371/journal.pone.0237145 (PMC7398551; doi:10.1371/journal.pone.0237145)
Supplement: Attachment — Submitted filename: Response_to_Reviewers.docx [file pone.0237145.s002.docx]

28 March 2020

Dear Dr. Scotti and the *PLOS One* reviewers:

Thank you for allowing us to revise the manuscript. The constructive suggestions and feedback provided by the reviewers have substantially improved our paper. Our revised manuscript contains modifications based on the reviewers’ suggestions.

Once again, thank you for your thorough and supportive peer review.

On behalf of the authors, yours sincerely,

Takashi Watari, MD, MCTM, MS

Corresponding author

**Reviewer #2:**

*“The authors have been reasonably responsive to the reviewer comments. They appear to have missed the point of the comment about separating claims that did and did not result in a payment, however. Because the authors’ aim is to say something about the nature of diagnostic errors using malpractice claims as a data source, their study question is more directly answered by examining claims that are meritorious (i.e. actually do involve an error) than by analyzing all claims, many of which (if data from several other countries are any indication) are nonmeritorious—i.e., do not actually relate to injuries due to an error. In the US, for example, estimates of the proportion of claims that are nonmeritorious range from a third to over half of all claims. That’s why it is especially useful, given the severe limitations of this data source, to isolate claims with the greatest signal about actual errors. Payment is an imperfect proxy measure of that; claims get paid sometimes even when they don’t involve errors. But it’s better than nothing.”*

**Response:**

We understand your comment for this study and wholly agree with your idea that it would be useful information for clinicians. Thus, we added the related information in response to the reviewer’s comment,

P7 L153

“In addition, we also analyzed the above 941 case (52.6%) of claims having a final judgement. Death was the most common claims outcome (n = 473/941; 50.27%), followed by sequelae (41.8%) and full recovery (6.3%) The median patient age was 32 years (interquartile range [IQR] = 10­.5–­­53), and 54.2% were men. The median claim duration was 7 years (M = 7.64 years, IQR = 5–9 years, maximum = 25 years). A total of 447 (47.5%, 95% confidence interval [CI]: 44.3%–50.7%) DERC cases were observed.”

However, our aim of this study was to better characterize the negative impact of diagnostic error reported in malpractice claims; hence, after this study, we will proceed with a comparison study in near future. We very much appreciate your constructive guidance.

**Reviewer #2:**

*“Some of the edits made to the manuscript read a bit rough; they could benefit from editing by a native English speaker and from the following revisions to the limitations section:”*

**Response:**

We thank the reviewer for this pertinent comment. Accordingly, we have changed the following text per the advice of a native-English-speaking editor:

**P6 L134 (only minor changes)**

“In the extracted data, malpractice claim frequency was measured using 10-year periods, and the number of claims in each time period was determined: before 1970 (n = 198; 11.0% of total malpractice claims); during the 1970s (n = 393; 21.8%); during the 1980s (n=366; 20.3%); during the 1990s (n = 623; 34.6%); during the 2000s (n = 182; 10.1%); and during the 2010s (n = 30; 1.7%). Although we collected all available malpractice claim cases from the web database, most data represented cases that occurred after the 1970s (n = 1,594; 88%). The DERC percentage for each 10-year period showed only the period before 1970, and the 2000s were significantly different (before 1970: 26.79%, p < 0.001; 2000s: 52.0%, p < 0.001). We also performed a multivariate logistic analysis to compare the DERC and non-DERC groups over each 10-year period and found no significant proportion of DERC among each 10-year period.”

*“- The new limitation added on p.18 (“there is no existing data on the frequency with which adverse events lead to malpractice claims in Japan”) should be called out as separate from the first limitation, as it is a wholly different point. This limitation should be labeled “Second,” and the limitations that followed should be renumbered accordingly.”*

and

*- The sentence beginning “Furthermore, there are possibly much more cases…” on p.19 does not belong where it is inserted. It is again a separate point, and it is the same point the authors already make farther down the page (“Third, many settlements…”).*

and

*- The sentence beginning “Furthermore, there are possibly much more cases…” on p.19 does not belong where it is inserted. It is again a separate point, and it is the same point the authors already make farther down the page (“Third, many settlements…”).*

Thank you for raising this important point. We completely agree with the reviewers’ comments; therefore, we have revised the text following these suggestions.

**P18 L294**

“Therefore, further training in general and emergency medicine as well as improvement of work conditions are required to minimize diagnostic errors in these settings in Japan.

Several limitations should be noted. First, although we used the largest claims database in Japan (similar to previous studies [12,29,32,33]), the data were not nationally representative of all malpractice claims. Second, there are no existing data on the frequency with which adverse events lead to malpractice claims in Japan. According to the Japanese Supreme Court report, the total number of adjudicated medical lawsuits in Japan, including those heard in brief and district courts, was not large. There were 305 malpractice claims that received their final judgments in 2000, 324 cases in 2010, and 269 cases in 2016. Third, the data from malpractice claims are not direct medical records and are thus not ideal sources for investigating error frequency and causal factors underlying such errors in an actual clinical setting. Nevertheless, to the best of our knowledge, this survey of claims contains detailed clinical information and is the largest such database in Japan. Fourth, our database only included information from claims in Japan, and it is difficult to generalize the present findings to other countries with different legal systems. Fifth, many cases where settlements were made both in and out of court may not have been included in this database.”

*-“Finally, the revisions do a good job overall of acknowledging that malpractice claims aren’t a very good source of information about all diagnostic errors, but they could be improved by revising a few remaining instances where the authors talk about errors rather than claims:”*

We would like to thank the reviewer for these kind comments. We apologize for being unclear about how representative the data are of all malpractice claims involving diagnostic errors in Japan. However, thanks to reviewer’s constructive suggestions, we have made these necessary changes to the manuscript.

*- P.16: “about 40% of the claims were considered diagnostic errors” should instead read “about 40% of the claims involved allegations of a diagnostic error”*

**P16 L242**

Our results based on 1,802 malpractice claims over a 56-year period in Japan showed that about 40% of the claims involved allegations of a diagnostic error

*- P.16: “The initial diagnoses most likely to lead to diagnostic errors were” should read “The diagnoses most commonly associated with allegations of diagnostic errors in malpractice claims were”*

**P16 L245**

The initial diagnoses most commonly associated with allegations of diagnostic errors in malpractice claims were upper respiratory tract infection (mainly common cold), non-bleeding digestive tract disease (mainly gastroenteritis), and “no abnormality.”

*- P.20: “diagnostic errors in the malpractice claims data from Japan were common” should instead read “diagnostic errors were a common allegation in malpractice claims”*

*- P.20: “and tended to result in poor patient outcomes” implies causality. It should instead read “and tended to involve allegations of relatively severe patient outcomes compared to other types of malpractice claims.”*

**P20 L329**

In our study, diagnostic errors were a common allegation in malpractice claims and tended to involve allegations of relatively severe patient outcomes compared to other types of malpractice claims.

Thank you again for your comments regarding our paper. I hope you find the revised manuscript suitable for publication.
