# Supplementary material for: Factors and impact of physicians’ diagnostic errors in malpractice claims in Japan
Source: PLoS One. 2020 Aug 3;15(8):e0237145. doi: 10.1371/journal.pone.0237145 (PMC7398551; doi:10.1371/journal.pone.0237145)
Supplement: Attachment — Submitted filename: Response_to_Reviewers.docx [file pone.0237145.s003.docx]

May 28 2020

Dear Dr. Scotti and the *PLOS One* reviewers:

Thank you for allowing us to revise the manuscript. The constructive suggestions and feedback provided by the reviewers have substantially improved our paper. Our revised manuscript contains modifications based on the reviewers’ suggestions.

Once again, thank you for your thorough and supportive peer review.

On behalf of the authors, yours sincerely,

Takashi Watari, MD, MCTM, MS

Corresponding author

**Reviewer #2:**

*“The authors have been reasonably responsive to the reviewer comments. They appear to have missed the point of the comment about separating claims that did and did not result in a payment, however. Because the authors’ aim is to say something about the nature of diagnostic errors using malpractice claims as a data source, their study question is more directly answered by examining claims that are meritorious (i.e. actually do involve an error) than by analyzing all claims, many of which (if data from several other countries are any indication) are nonmeritorious—i.e., do not actually relate to injuries due to an error. In the US, for example, estimates of the proportion of claims that are nonmeritorious range from a third to over half of all claims. That’s why it is especially useful, given the severe limitations of this data source, to isolate claims with the greatest signal about actual errors. Payment is an imperfect proxy measure of that; claims get paid sometimes even when they don’t involve errors. But it’s better than nothing.”*

**Response:**

I wholeheartedly agree with your view that the comparison of meritorious trials alone would provide the most useful answer for our study question, and we have to apologize for the misunderstanding caused by the contextual differences between Japanese and US legal constructs. For example, “Claims with final judgment resulting in payment” (Table 1 in the revised manuscript) refers to cases where judgment was in favor of the patient, resulting in a payment order for the medical practitioner. We have revised the manuscript to clarify our meaning.

I do believe there is a great difference between the way medical litigation is practiced in the US compared to Japan. There are only about 800 medical litigation cases in Japan each year, most of which are meritorious. While we realize that cultural practices cannot be quantified as evidence, it is important to note that Japanese people are generally reluctant to dispute healthcare-related decisions. This means that trials usually only result from cases where there is an obvious problem in the care provided by medical staff. On the other hand, the number of medical litigation cases in the US is about 17,000 a year – 21 times more than the number of cases in Japan. Furthermore, the number of medical litigation cases per person is 7.7 times more in the US, compared to Japan. Considering these contextual differences, if this study was conducted in the US and included all medical litigation, the data would include an overwhelmingly high number of unmeritorious cases, and be strongly biased. All the cases in our dataset had been concluded, and clarified the validity of claims in detail. We do not consider those cases not resulting in payment to be unmeritorious, and we conclude that there are currently few unmeritorious medical cases under our judicial system.

Second, under the supervision of the third author, a lawyer, we narrowed our dataset down to 1,802 meritorious medical cases that deserve analysis (Figure 1). This work took an enormous amount of time and ruled out any non-meritorious trials to eliminate bias as much as possible. Notably, of the approximately 1,900 medical litigation cases, only 34 were classified as unjustified and unjust by the third author. As noted previously, unfair trials in medical litigation are rare in Japan.

Third, the frequency of doctors’ diagnostic errors in actual medical practice is high. In other words, if we only analyze the case when the doctor loses and payment is required, the actual diagnostic error leading to the winning lawsuit would be ignored, even though the doctor had made a diagnostic error. Our concern was that we would exclude winning cases where diagnostic errors existed, as medical errors are difficult to prove in Japanese medical law, resulting in low-cost medical professional liability insurance; thus, even when clear diagnostic errors exist, it would not necessarily lead to medical litigation.

I believe your opinion is correct in the context of US medical law, and our next goal is to conduct a second analysis based on your advice. I really appreciate your constructive peer review.

**Changes**

P7 L153

“In addition, we specifically analyzed the 941 claims where final judgment resulted in payment. Among these, death was the most common claim outcome (n = 473/941; 50.27%), followed by sequelae (41.8%) and full recovery (6.3%). The median patient age was 32 years (interquartile range [IQR] = 10­.5–­­53), and 54.2% were men. The median claim duration was 7 years (M = 7.64 years, IQR = 5–9 years, maximum = 25 years). Of these, 447 claims (47.5%, 95% confidence interval [CI]: 44.3%–50.7%) were DERC cases. ”

**Reviewer #2:**

*“Some of the edits made to the manuscript read a bit rough; they could benefit from editing by a native English speaker and from the following revisions to the limitations section:”*

**Response:**

We thank the reviewer for this pertinent comment. Accordingly, we have revised the following text per the advice of a native-English-speaking editor:

**(only minor changes)**

**P6 L133**

“In the extracted data, malpractice claim frequency was measured using 10-year periods, and the number of claims in each period was determined: before 1970 (n = 198; 11.0% of total malpractice claims), during the 1970s (n = 393; 21.8%), during the 1980s (n=366; 20.3%), during the 1990s (n = 623; 34.6%), during the 2000s (n = 182; 10.1%), and during the 2010s (n = 30; 1.7%). Although we collected all available malpractice claim cases from the database, most data represented cases that occurred after the 1970s (n = 1,594; 88%). The DERC percentage for each 10-year period was significantly different only for the period before 1970 and during the 2000s (before 1970: 26.79%, p < 0.001; 2000s: 52.0%, p < 0.001). We also performed a multivariate logistic analysis to compare the DERC and non-DERC groups over each 10-year period and found no significant proportion of DERC for any period.”

**Reviewer #2:**

*“- The new limitation added on p.18 (“there is no existing data on the frequency with which adverse events lead to malpractice claims in Japan”) should be called out as separate from the first limitation, as it is a wholly different point. This limitation should be labeled “Second,” and the limitations that followed should be renumbered accordingly.”*

and

*- The sentence beginning “Furthermore, there are possibly much more cases…” on p.19 does not belong where it is inserted. It is again a separate point, and it is the same point the authors already make farther down the page (“Third, many settlements…”).*

Thank you for raising these important points. We completely agree with the reviewers’ comments; therefore, we have revised the text following these suggestions.

**Changes**

**P18 L294**

“Therefore, further training in general and emergency medicine, along with improvement of work conditions, are required to minimize diagnostic errors in these settings in Japan. However, several limitations of this study should be noted. First, although we used the largest claims database in Japan (similar to previous studies [12,29,32,33]), the data were not nationally representative of all malpractice claims. Second, there are no existing data on the frequency with which adverse events lead to malpractice claims in Japan. According to the Japanese Supreme Court report, the total number of adjudicated medical lawsuits in Japan, including those heard in brief and district courts, was not large [13]: there were 305 malpractice claims that received their final judgments in 2000, 324 in 2010, and 269 in 2016. Third, the data from malpractice claims are not direct medical records and are thus not ideal sources for investigating error frequency and causal factors underlying such errors in an actual clinical setting. Nevertheless, to the best of our knowledge, this survey of claims contains detailed clinical information and is the largest such database in Japan. Fourth, our database only included information from claims in Japan, and it is difficult to generalize the present findings to other countries with different legal systems. Fifth, many cases where settlements were made both in and out of court may not have been included in this database.

**Reviewer #2:**

*-“Finally, the revisions do a good job overall of acknowledging that malpractice claims aren’t a very good source of information about all diagnostic errors, but they could be improved by revising a few remaining instances where the authors talk about errors rather than claims:”*

We would like to thank the reviewer for these kind comments. We apologize for being unclear about how representative the data are of all malpractice claims involving diagnostic errors in Japan. However, thanks to the reviewer’s constructive suggestions, we have made necessary changes to the manuscript.

**Changes**

**P16 L244**

Our results, based on 1,802 malpractice claims over a 56-year period in Japan, showed that about 40% of the claims involved allegations of a diagnostic error, that

**Reviewer #2:**

*- P.16: “The initial diagnoses most likely to lead to diagnostic errors were” should read “The diagnoses most commonly associated with allegations of diagnostic errors in malpractice claims were”*

**Changes**

**P16 L24**

The initial diagnoses most commonly associated with allegations of diagnostic errors in malpractice claims were upper respiratory tract infection (mainly the common cold), non-bleeding digestive tract disease (mainly gastroenteritis), and “no abnormality.”

**Reviewer #2:**

*- P.20: “diagnostic errors in the malpractice claims data from Japan were common” should instead read “diagnostic errors were a common allegation in malpractice claims”*

*- P.20: “and tended to result in poor patient outcomes” implies causality. It should instead read “and tended to involve allegations of relatively severe patient outcomes compared to other types of malpractice claims.”*

**Changes**

**P20 L328**

In our study, diagnostic errors were a common allegation in malpractice claims and tended to involve allegations of relatively severe patient outcomes compared to other types of malpractice claims.

Thank you again for your feedback on our paper. I hope you find the revised manuscript suitable for publication.
